# Evaluation of three enzyme-linked immunosorbent assays for sarcoptic mange diagnosis and assessment in the Iberian ibex, *Capra pyrenaica*

**DOI:** 10.1186/s13071-016-1843-4

**Published:** 2016-10-21

**Authors:** Arián Ráez-Bravo, José Enrique Granados, Emmanuel Serrano, Debora Dellamaria, Rosa Casais, Luca Rossi, Anna Puigdemont, Francisco Javier Cano-Manuel, Paulino Fandos, Jesús María Pérez, José Espinosa, Ramón Casimiro Soriguer, Carlo Citterio, Jorge Ramón López-Olvera

**Affiliations:** 1 Servei d’Ecopatologia de Fauna Salvatge (SEFaS), Wildlife Health Service - Departament de Medicina i Cirurgia Animal, Universitat Autònoma de Barcelona, Bellaterra, Barcelona E-08193 Spain; 2 Espacio Natural Sierra Nevada, Carretera Antigua de Sierra Nevada, Km 7, E-18071 Pinos Genil, Granada, Spain; 3 Departamento de Biologia & CESAM, Universidade de Aveiro, Aveiro, Portugal; 4 Istituto Zooprofilattico Sperimentale delle Venezie, Legnaro, PD Italy; 5 Servicio Regional de Investigación y Desarrollo Agroalimentario (SERIDA), Centro de Biotecnología Animal, La Olla-Deva, E-33394 Asturias, Spain; 6 Dipartimento di Scienze Veterinarie, Università di Torino, Torino, Italy; 7 Departament de Farmacologia, Facultat de Veterinària, Universitat Autònoma de Barcelona, Barcelona, Bellaterra Spain; 8 Agencia de Medio Ambiente y Agua, Isla de la Cartuja, E-41092 Sevilla, Spain; 9 Departamento de Biología Animal, Biología Vegetal y Ecología, Universidad de Jaén, Campus Las Lagunillas, s.n, E-23071 Jaén, Spain; 10 Estación Biológica de Doñana (CSIC), Av. Américo Vespucio, s.n, E-41092 Sevilla, Spain

**Keywords:** *Capra pyrenaica*, Diagnostic, ELISA, *Sarcoptes scabiei*, Serum

## Abstract

**Background:**

Sarcoptic mange is a contagious skin disease caused by the mite *Sarcoptes scabiei*, affecting different mammalian species worldwide including the Iberian ibex (*Capra pyrenaica*), in which mortalities over 90 % of the population have been reported. No efficient diagnostic methods are available for this disease, particularly when there are low mite numbers and mild or no clinical signs. In this study, three enzyme-linked immunosorbent assays (ELISA) developed for dog (ELISA A), Cantabrian chamois (*Rupicapra pyrenaica parva*) (ELISA B) and Alpine chamois (*Rupicapra rupicapra*) (ELISA C), were evaluated to detect specific antibodies (IgG) to sarcoptic mange in Iberian ibex sera.

**Methods:**

Serum samples from 131 Iberian ibexes (86 healthy and 45 scabietic) were collected from 2005 to 2012 in the Sierra Nevada Natural and National Parks (southern Spain). Based on visual inspection, ibexes were classified into one of three categories, namely healthy (without scabietic compatible lesions), mildly affected (skin lesions over less than 50 % of the body surface) and severely affected (skin lesions over more than 50 % of the body surface). The optimal cut-off point, specificity, sensitivity and the area under the curve (AUC) were calculated, and the agreement between tests was determined. Moreover, differences in the optical density (OD) related to scabies severity have been evaluated for the best test.

**Results:**

ELISA C showed better performance than the two other tests, reaching higher values of sensitivity (93.0 %) and specificity (93.5 %) against the visual estimation of the percentage of affected skin, chosen as the gold standard. Significantly higher concentrations of specific antibodies were observed with this test in the mildly and severely infested ibexes than in healthy ones.

**Conclusions:**

Our results revealed that ELISA C was an optimal test to diagnose sarcoptic mange in the Iberian ibex. Further studies characterizing immune response during the course of the disease, including spontaneous or drug induced recovery, should follow in order to better understand sarcoptic mange in Iberian ibex populations.

## Background

Sarcoptic mange is a contagious skin disease caused by the mite *Sarcoptes scabiei* (Linnaeus, 1758). This parasite is worldwide distributed [1–3], causing disease in a wide range of mammalian species [2]. Wild ungulates are one of the most affected groups, as demonstrated by mange outbreak records in African and European ungulates [4–6]. All wild ruminant species in Spain have also been affected by sarcoptic mange, even causing fatal epizootics, e.g. Iberian ibex (*Capra pyrenaica*) [7–9], Cantabrian chamois (*Rupicapra pyrenaica parva*) [10] and aoudad (*Ammotragus lervia*) [11]. The clinical signs and lesions in individuals infected by *S. scabiei* are characterized by pruritus, drying, peeling, alopecia, hyperkeratosis and scab formation. In population terms, this parasite is able to cause severe demographic downturns, up to above 90 % on occasion [12]. In fact, stochastic simulations on population extinction have shown that the impact of sarcoptic mange on ungulate populations can be comparable to the impact observed for emerging viral diseases [13].

Iberian ibex is a mountain ungulate endemic to the Iberian Peninsula [14]. Andalusia populations (southern Spain) have suffered sarcoptic mange outbreaks since 1987 [7], and recently the eastern populations have been also affected by this disease. Different methods exist for the diagnosis of sarcoptic mange, although none of them showed the ideal specificity and sensitivity [15]. Amongst direct diagnostic methods, the gold standard is the microscopical detection of mites, exuviae, eggs or faeces in scrapings of infested skin. Although this method is deemed 100 % specific, low sensitivity was found at low mite densities [15, 16]. Visual diagnosis of scabies compatible lesions has also been used for monitoring the disease in free-ranging ibexes [17], and this disease has been included among those to check before translocating wild ruminants in Spain, visual inspection being the legally mandatory diagnostic method [18]. A number of methods have been developed or proposed in an attempt to overcome such diagnostic deficiency, including the adhesive tape test [16, 19], serological methods [20, 21], polymerase chain reaction (PCR) [22, 23], dermoscopy [16, 24, 25], termography [26], or trained disease-detector dogs [27].

*Sarcoptes scabiei* stimulate E and G immunoglobulin production in infested hosts [28–33], including Iberian ibex [34, 35]. Different commercial enzyme-linked immunosorbent assays (ELISA) have been evaluated to detect specific antibodies to *S. scabiei* in dogs [36, 37], pigs [38, 39], wild boar [40] or chamois [41]. However, the use of ELISA tests to diagnose scabies in Iberian ibex has not yet been evaluated.

In this study, three IgG indirect ELISA tests were compared as diagnostic tools of sarcoptic mange in ibexes showing different lesional severity. In particular, the objectives of this study are: (i) to estimate the optimal cut-off points, specificity and sensitivity of the ELISA tests in Iberian ibex; (ii) to determine the agreement between tests; and (iii) to determine if a correlation between mange severity and the detected levels of humoral immune response (IgG) exists.

## Methods

### Sample collection

Serum samples from 131 healthy and scabietic Iberian ibexes were collected from 2005 to 2012 in Sierra Nevada Natural and National Parks (36°00'–37°10'N, 2°34'–3°40'W) (Table 1). The ibexes were chemically immobilized using a combination of ketamine and xylazine (3.0 + 3.0 mg/kg) [42]. After induction, blood samples were collected from the jugular vein and kept at 4 °C in a cold box. The age of the ibexes was estimated by horn-segment counts [12], and ranged between 1 and 12 years. Based on the visual estimation of the percentage of scabietic skin, each individual was classified into one of three categories [17, 43]: healthy (although the authors are aware that the absence of scabietic-compatible lesions is not a synonym of “uninfested”, for the purpose of this study “healthy” will be used as “without visible scabietic-compatible lesions”), mildly infested (0–50 % of the body surface affected by sarcoptic mange), and severely infested (more than 50 % of the surface affected) (Table 2). This diagnostic method was considered as the “gold standard” to evaluate the three ELISA tests.

**Table 1:** Number of Iberian ibexes (*Capra pyrenaica*) sampled in the Sierra Nevada Natural and National Parks according to their sex and sarcoptic mange status

Sarcoptic mange status	Sex		Total
	Males	Females	
Healthy^a^	59	27	86
Mildly infested^b^	31	1	32
Severely infested^c^	9	4	13
Total	99	32	131

^a^Without scabietic compatible lesions

^b^Less than 50 % of the body surface affected

^c^More than 50 % of the body surface affected

**Table 2:** Number of Iberian ibexes (*Capra pyrenaica*) sampled in the Sierra Nevada Natural and National Parks according the ELISA results and the categories based on the visually estimated percentage of affected skin [17]

		Healthy^a^	Infested		
			Mildly^b^	Severely^c^	Total
ELISA A	Positive serology	9	10	4	14
	Negative serology	76	21	8	29
	Total	85	31	12	43
ELISA B	Positive serology	16	13	12	25
	Negative serology	58	17	1	18
	Total	74	30	13	43
ELISA C	Positive serology	5	27	13	40
	Negative serology	72	3	0	3
	Total	77	30	13	43

^a^Without scabietic compatible lesions

^b^Less than 50 % of the body surface affected

^a^More than 50 % of the body surface affected

### Serological analyses

Blood samples were allowed to clot at room temperature. Within 24 h from collection, serum was obtained by centrifugation at 4,750× *g* for ten minutes and stored at -20 °C until analysis.

The Iberian ibex sera were tested by three indirect ELISAs developed for dog (ELISA A, *n* = 128) [44], Cantabrian chamois (ELISA B, *n* = 117) [45] and Alpine chamois (ELISA C, *n* = 120) (in-house indirect ELISA, Istituto Zooprofilattico Sperimentale delle Venezie, modified from [41]). ELISA A is a commercial ELISA (Univet, Barcelona, Spain) developed for the diagnosis in dog. The plates are coated with *S. scabiei* var. *canis* antigen. To adapt the ELISA A for its use in ibex, specific antibodies for goat (Donkey antigoat IgG-HRP SC-2020, Santa Cruz Biotechnology, Dallas, Texas, USA) were used. ELISA B is based on a structural antigen of the mite (Ssλ20∆B3), whose encoding cDNA was identified by screening of *S. scabiei* var. *hominis* library using the sera from an infected chamois, and expressed in *Escherichia coli* as a unitary recombinant antigen [45]. ELISA C is an in-house method, developed by the Istituto Zooprofilattico Sperimentale delle Venezie and validated for lung extract. ELISA C is a modification from [41] by using commercial ELISA plates coated with *S. scabiei* var. *suis* antigen (Sarcoptes-Elisa 2001® PIG, AFOSA GmbH, Blankenfelde-Mahlow, Germany) instead of the red fox (*Vulpes vulpes*) *S. scabiei* antigens originally used [41]. This test uses the avidin-biotin detection system, as previously reported [41].

Samples were analyzed in duplicate, and in triplicate in ELISA C.

The optical density (OD) was read at 450 nm in ELISA A and B, and at 405 nm in ELISA C, and was expressed as Optical Density percentage (OD %) using the following formula:

$$ OD\%=\frac{\left(ODS- ODNcon\right)}{\left( ODPcon- ODNcon\right)}\times 100 $$

where OD % is the optical density percentage; ODS is the mean optical density (OD) of the sample (two or three replicates); ODNcon is the mean OD of the negative control; and ODPcon is the mean OD of the positive control.

Ten sera from healthy Iberian ibexes (animals without skin lesions) without previous contact with mange were used as negative controls, whereas ten sera from actively sarcoptic mange-infested Iberian ibexes were used as positive controls. The same negative and positive control sera were used for the three ELISA tests, and all controls consistently showed low (negative controls) and high (positive controls) values for the three ELISA tests.

### Statistical analysis

The optimal cut-off points between positive and negative samples was estimated using the Youden index, maximizing the difference between true positive rate (sensitivity) and false positive rate (1 - specificity). Thereby, the maximum of sensitivity and specificity is achieved [46, 47]. Once the optimal cut-off points were established and positive and negative individuals assigned, the specificity and sensitivity of the three ELISA tests were then established using the following formulae [48]:

$$ \begin{array}{c}\hfill Specificity=\frac{true\kern0.5em  negative\kern0.5em  animals}{true\kern0.5em  negative\kern0.5em  animals+ false\kern0.5em  positive\kern0.5em  animals}\times 100\hfill \\ {}\hfill Sensitivity=\frac{true\kern0.5em  positive\kern0.5em  animals}{true\kern0.5em  postive\kern0.5em  animals+ false\kern0.5em  negative\kern0.5em  animals}\times 100\hfill \end{array} $$

After that, the area under a receiver operating characteristic (ROC) curve, a graph of true positive rate (sensitivity) *versus* false positive rate (1 - specificity), was used to determine the diagnosis accuracy of the ELISA tests [49, 50].

The agreement between the ELISA tests was evaluated by the Cohen’s Kappa coefficient [51] and Bland-Altman plots [52, 53]. The Cohen’s Kappa coefficient ranges between 0 and 1, and values close to 1 indicate high correlation. Bland-Altman plot describes the agreement between two quantitative measurements by constructing limits of agreement, which are calculated by using the mean and the standard deviation of the differences between two measurements. The difference of the two paired measurements is plotted against the mean of the two measurements.

Since ELISA results did not fit a normal distribution, as assayed by Agostino skewness test and Bonett-Seier test for Geary kurtosis, Kruskal-Wallis with Mann-Whitney tests with Bonferroni correction were used to evaluate whether optical density (a proxy for immunoglobulin G production) was influenced by mange severity (healthy, mildly and severely infested). This analysis was performed only with the optical density values from the ELISA test showing the highest sensitivity and specificity against the gold standard (ELISA C, see below).

Although sex and season determine sarcoptic mange severity and progression in Iberian ibex [17, 34, 54, 55], the effects of these two factors on ELISA performance could not be evaluated due to insufficient sample size. Statistical analyses were performed using the R software version 3.2.4 (R Development Core Team 2016, The R Foundation, Vienna, Austria). Skewness and kurtosis tests were performed with the “*moments*” package [56], Bland-Altman regressions with the “*BlandAltmanLeh*” package [57], the Kappa coefficients with the “*irr*” package [58], and the ROC-curves and the cut-off points were estimated with the “*OptimalCutpoints*” package [59].

## Results

**Fig. 1:**
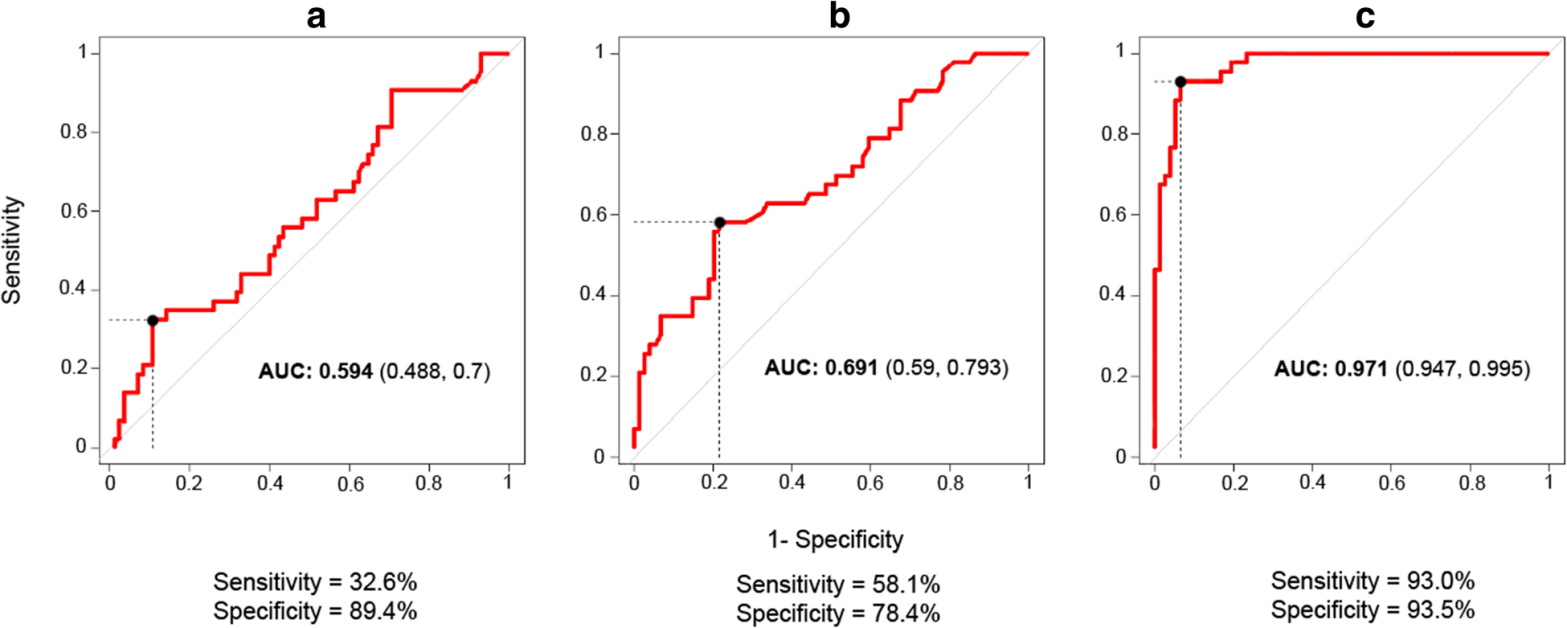
Receiver operating characteristic (ROC) curves for ELISA A (**a**), ELISA B (**b**) and ELISA C (**c**) for the detection of antibodies against *Sarcoptes scabiei* in Iberian ibex. The *red* line shows the mean area under the curve (AUC) plot, with the AUC value and the 95 % confidence intervals in parentheses. The sensitivity and specificity values correspond to the points in the plots

The cut-off points, sensitivity and specificity obtained for the three ELISA tests are shown in Table 3. The best results were obtained with ELISA C (cut-off = 92.0 % OD; sensitivity = 93.0 %; specificity = 93.5 %), followed by ELISA B (cut-off = 10.4 % OD; sensitivity = 58.1 %; specificity = 78.4 %) and ELISA A (cut-off = 45.5 % OD; sensitivity = 32.6 %; specificity = 89.4 %). Consequently, the area under the curve (AUC) obtained for ELISA C was close to 1 (AUC = 0.971), indicating an excellent diagnostic accuracy. Conversely, the AUC for ELISA A and B was lower (0.594 and 0.691, respectively), indicating a poorer diagnosis accuracy of these two tests (Fig. 1, Table 3). The false negative ibexes (i.e. ibexes with negative ELISA results but showing skin lesions compatible with sarcoptic mange) belonged to the mildly infested category in all cases (100 %, 3 out of 3) for ELISA C and most cases for ELISA B (94.4 %, 17 out of 18) and ELISA A (72.4 %, 21 out of 29) (Table 2). Furthermore, the three mildly infested false negative cases for ELISA C, 15 out of 17 for ELISA B, and 13 out of 21 for ELISA A had less than 25 % of the body surface affected, based on the visually estimated percentage of affected skin [17, 43].

**Table 3:** Area under the curve (AUC) and its 95 % confidence interval (CI), cut-off, sensitivity and specificity values of the ELISA tests

	ELISA A	ELISA B	ELISA C
AUC (95 % CI)	0.594 (0.488–0.700)	0.691 (0.590–0.793)	0.971 (0.947–0.995)
Cut-off (% OD)	45.5	10.4	92.0
Sensitivity (%)	32.6	58.1	93.0
Specificity (%)	89.4	78.4	93.5

**Fig. 2:**
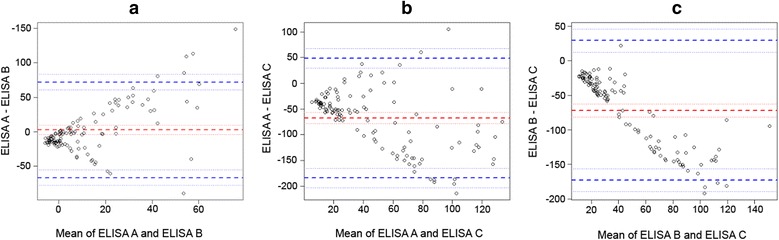
Bland-Altman plots comparing the ELISA tests. *Dashed blue lines* indicate the confidence interval limits for the agreement limits (± 1.96*standard deviation); *dashed red lines* indicate the confidence interval limits for the mean bias difference. **a** Comparison of results for ELISA A and ELISA B: differences tended to increase with increasing mean % OD values. **b** Comparison of results for ELISA A and ELISA C: differences tended to decrease with increasing mean % OD values. **c** Comparison of results for ELISA B and ELISA C: differences decreased with increasing mean % OD values

Bland-Altman plots show that the three ELISA tests are not interchangeable, especially at high values of OD (Fig. 2). Along the same lines, the Cohen’s Kappa coefficient (*k*) indicated that there is not a good strength of agreement between the three tests. The ELISA B and C showed the higher strength of agreement, which was only moderate, whereas the strength of agreement between the ELISA A and C was still lower (fair) and the ELISA A and B showed the lowest strength of agreement (slight) [60].

The relationship between sarcoptic mange severity and the % OD values was analyzed only for ELISA C, due to the better performance of this test as compared with the other two (Table 4). Significant differences in the % OD (Kruskal-Wallis test: *χ* ^2^ = 73.99, *df* = 2, *P* < 0.0001) among the three categories of sarcoptic mange status were found. The healthy ibexes showed statistically significant lower % OD values than the mildly (*W* = 90, *P* < 0.0001) and severely (*W* = 6, *P* < 0.0001) infested ibexes. The difference between the higher % OD of the severely infested ibexes and the lower % OD of the mildly infested ones was close to significance (*W* = 106, *P* = 0.058).

**Table 4:** ELISA results (% OD) based on a visually estimated percentage of affected skin [17] in Iberian ibex (*Capra pyrenaica*) sampled in the Sierra Nevada Natural and National Parks

	Healthy			Mildly infested			Severely infested		
	*n*	Mean	SD	*n*	Mean	SD	*n*	Mean	SD
ELISA A	85	9.50	28.55	31	19.46	33.49	12	24.28	41.43
ELISA B	74	7.63	9.45	30	13.76	19.61	13	34.71	28.72
ELISA C*	77	47.61^a^	26.45	30	137.86^b^	41.59	13	168.16^b^	25.62

*Kruskal-Wallis test: *χ* ^2^ = 73.99, *df* = 2, *P* < 0.0001; ^a,b^ means with ﻿different superscripts are statistically different

*Abbreviation*: *SD* standard deviation

## Discussion

This study compared three ELISA tests for the detection of specific serum antibodies to *S. scabiei* in the Iberian ibex, which showed different performance in terms of specificity and sensitivity. Furthermore, the OD % varied among degrees of sarcoptic mange infestation for the best performing test.

To the best of our knowledge, this is the first time in which several ELISA tests are compared and evaluated for the diagnosis of sarcoptic mange in Iberian ibex. Based on results of this study, ELISA C was the best performing test to detect specific antibodies against *S. scabiei* in this species, showing higher sensitivity and specificity and an AUC close to 1. This test was validated for the detection of IgG in Alpine chamois lung extracts with a sensitivity of 100 % and a specificity of 98.6 % (data not published). Moreover, the ELISA C allowed the differentiation of healthy and mangy ibexes. ELISA B revealed lower specificity and sensitivity than previously reported in Cantabrian chamois and red deer, with a specificity of 97 %, and a sensitivity of 100 % and 75 % in chamois and red deer [45, 61], respectively. ELISA A had never been used before this study for the detection of antibodies against *S. scabiei* in wild ungulates, and the sensitivity and specificity of the test in dog are 92.1 % and 94.6 %, respectively.

The avidin-biotin detection system used in the ELISA C could be one of the reasons explaining the best performance of ELISA C as compared to ELISAs B and C. This method has the potential to increase the sensitivity of the indirect ELISA, since avidin has four binding sites for biotin, resulting in an essentially irreversible complex which is stable in later washes and incubations [62]. Although serum cross-reactivity against different *S. scabiei* strains exists, each host species responds differently to different mite varieties [63, 64]. Therefore, the differences in sensitivity among the three ELISA tests could also depend on the specific antigens used and the origin of mites.

False negative results (i.e. mangy ibexes in which no antibodies against *S. scabiei* were detected) could be explained by low levels of circulating IgG in recently infested ibexes or in chronic infestations. The clinical signs of sarcoptic mange may appear before IgG detection in different species [29, 30, 32, 38, 65], and in experimentally infected Iberian ibex, IgG were detected no earlier than 18−27 days post-infestation [34]. This time lapse may explain at least some of the false negative ibexes within the mildly infested category. On the other hand, maximum OD values indicating specific IgG peaks occur between 50 days and 12–16 weeks post infestation in domestic goats, pigs and Iberian ibexes, to decline slightly or plateauing afterwards [20, 32, 34]. This could explain some false negative results in chronically affected ibexes.

A low test specificity (i.e. positive ELISA results in healthy ibexes) can be explained by a lack of detection of minute skin lesions [17, 40], subclinical infestations [66], or cross reactions with antigens from other related parasites [67, 68]. Cross-reactivity against dust mites, ticks and *Trombicula* spp., a mite that affects the skin of different European wild ungulates like chamois [69], have been previously assessed and discarded for ELISA A, B and C, respectively [41, 44, 45]. Therefore, false positive results could be putatively related to cross-reactions with other parasites reported to subclinically infest wild Iberian ibex, such as other mites (*Psoroptes* sp., *Trombicula* sp.) [70] or different tick species [70, 71]. ELISA C showed lower specificity than previously reported in Alpine chamois probably due to the application of the test on two different types of samples with different antibodies amount (serum *versus* lung extracted). However, false positive ibexes could also be due to individuals having had previous contact with *S. scabiei* and having recovered from the clinical phase of the disease. Hosts infested with *S. scabiei* develop resistance to re-infestation [28] and healthy Iberian ibexes exposed to sarcoptic mange show detectable IgG levels [34, 35]. In Sierra Nevada, sarcoptic mange is endemic, thus some of the healthy Iberian ibexes of this study could have had previous contacts with the mite, thus developing an IgG response. However, the higher *S. scabiei*-positivity by ELISA in the severely infested ibexes suggests that IgGs are not protective against mange. In general, protection against mange has been associated with a cell-mediated immune response as well as with a humoral immune response [28, 29, 32, 65, 72, 73]. Therefore, further research appears to be needed to understand the specific role of both humoral and cellular immune responses. The significant differences in % OD found between healthy and infested ibexes for ELISA C are not consistent with a previous report, in which there were no % OD differences between infested and healthy ibexes from the same area [35]. However, results in this study fit the differences in two acute phase proteins (APP), namely alpha-1 acid glycoprotein (AGP) and serum amyloid A (SAA), observed in similarly ranked Iberian ibex. The higher APP level in severely affected ibexes was attributed to skin inflammation or the pathological secondary amyloidosis, leading to organ dysfunction in this category [74]. The increasing % OD values with increasing sarcoptic mange severity found in this study could be explained by a higher intensity of infestation in severely affected ibexes, which in turn would stimulate more intensely the immune system and elicit a higher antibody production. Scabies-specific IgG rapidly decline in parallel with the disappearance of mites from the skin in goats [32], and the percentage of mange-damaged skin and mite load are related in Iberian ibex [17]. Moreover, although in some species skin lesions are reflective of delayed hypersensitivity reaction associated with few or no mites, in Iberian ibex mites are definitely abundant in the skin in the generalized stage of the disease [2]. Therefore, the % OD results obtained with ELISA C test would not only indicate the presence of viable *S. scabiei* in the skin, but could also correlate with mite load.

The evaluation of the ELISA tests carried out in this study and the identification of ELISA C as a reliable tool for sarcoptic mange diagnosis in Iberian ibex will be useful in future research. Nevertheless, the limitations in sensitivity and specificity should be considered while assessing the results, and more studies on (i) the dynamics of the antibody response after *S. scabiei* infestation in Iberian ibex; (ii) the correlation between parasitic load, mange severity and immune response; and (iii) the influence of sex [34, 54] and season [17, 55] on the OD % are necessary in order to better understand the immune response to sarcoptic mange in the Iberian ibex.

## Conclusions

In conclusion, the ELISA developed for diagnose sarcoptic mange in Alpine chamois (Istituto Zooprofilattico delle Venezie, data not published) has been validated in this study as an effective test to be similarly applied in Iberian ibex. This can be helpful for completing the legal requirements for the transport of wild ungulates [18] and for seroepidemiological studies.
